# Treatment of Liver Metastases in Patients with Neuroendocrine Tumors of Gastroesophageal and Pancreatic Origin

**DOI:** 10.1155/2012/131659

**Published:** 2012-02-29

**Authors:** Ping Gu, Jennifer Wu, Elliot Newman, Franco Muggia

**Affiliations:** ^1^Department of Hematology and Medical Oncology, NYU Cancer Institute, New York, NY 10016, USA; ^2^Department of General Surgery, NYU Medical Center New York, NY 10016, USA; ^3^Department of Medical Oncology, NYU Cancer Institute, New York, NY 10016, USA

## Abstract

Well-to-moderately differentiated neuroendocrine tumors of gastroesophageal and pancreatic origin (GEP-NETs) with liver metastasis are a heterogeneous group of malignancies for which a range of therapeutic options have been employed. Surgical resection of hepatic metastases or hepatic artery embolization may be beneficial in patients with hepatic-predominant metastatic disease. Patients with “carcinoid” syndrome and syndromes associated with functional pancreatic NET (PNET) can be effectively treated with somatostatin analogs. On the other hand, the efficacy of systemic chemotherapy for these patients is limited. A placebo-controlled, double-blind, prospective, and randomized study showed that octreotide LAR improves progression-free survival in patients with advanced midgut functional “carcinoids.” In patients with advanced pancreatic NET, randomized, placebo-controlled studies have recently demonstrated that treatment with the tyrosine kinase inhibitor sunitinib or with mTOR inhibitor everolimus is associated with improved progression-free survival. Based on these studies, octreotide LAR, sunitinib, or everolimus are now considered as first-line therapeutic options in patients with advanced NET. Future studies will likely further define the role of these agents in patients with carcinoid liver metastasis and pancreatic NET liver metastasis.

## 1. Introduction

Neuroendocrine tumors of gastroesophageal and pancreatic origin (GEP-NETs) are a heterogeneous group of tumors characterized by their secretion of hormones or vasoactive peptides often resulting in specific hormone hyperfunction syndromes. NETs have recently been shown to be more common than previously suspected. In an analysis of the Surveillance, Epidemiology, and End Results (SEER) database, the estimated age-adjusted annual incidence of NET in 2004 was 5.25 per 100,000 people [1].

The prognosis and management of GEP-NETs is guided by histological classification. As a general rule, tumors with a high grade (grade 3), a mitotic count of more than 20 per 10 high-powered fields, or a Ki-67 proliferation index of more than 20% represent highly aggressive malignancies with a short clinical course and poor survival outcomes. The approach for these tumors is similar to that for small cell lung cancer and is not discussed in this review.

The focus of this review is the well-to-moderately differentiated GEP-NETs. These tumors can be subclassified into two general categories: pancreatic neuroendocrine tumors (PNETs) and others, mostly arising in the intestine, and often associated with the “carcinoid” syndrome. The terminology of “endocrine tumor” is replacing “neuroendocrine tumor” in pancreatic neuroendocrine tumors. And carcinoid is often linked to the secretion of serotonin and other vasoactive peptites resulting in “carcinoid” syndrome. This syndrome is manifested by episodic flushing, wheezing, diarrhea, and eventual right-sided valvular heart disease. Syndromes associated with hormone-secreting PNET can be manifested in insulinoma, glucagonoma, vasoactive intestinal peptide (VIP)-oma, and gastrinoma.

The majority of PNET occurs sporadically, but these tumors can also belong to a number of inherited syndromes associated with mutations in well-studied oncogenes and tumor suppressor genes. These syndromes include multiple endocrine neoplasia (MEN) types 1 and 2, von Hippel-Lindau disease, and tuberous sclerosis [2]. Patients with NET with such syndromes may represent subgroups particularly responsive to novel therapies targeting the underlying genetic defect or pathway.

GEP-NETs typically have an indolent natural history, even in the setting of metastasis. The treatment of patients with localized NET is primarily surgical. There are no data that demonstrate a benefit associated with adjuvant therapies. However, GEP-NETs commonly metastasize to liver, with up to 44% of patients developing neuroendocrine liver metastasis (NELM) over the course of their disease [3]. This review summarizes the current approach to NELM based on clinical trials in the past 10 years, emphasizing the differences between NELM arising from carcinoid and those from PNET.

## 2. Locoregional Therapies

### 2.1. Surgical Resection

Symptom control and improved quality of life and overall survival can be achieved by the reduction of circulating hormone levels via functional hormonal blockade, transarterial chemoembolization (TACE), or radiofrequency thermal ablation (RFA), thus obviating surgery. However, hepatic resection is often considered in patients with limited hepatic disease. If more than 90% of the tumor mass can be removed, these patients have an outcome similar to those with complete resection (resection of all visible hepatic tumors) [4, 5].

Mayo et al. [6] reported the outcomes of 339 patients from 8 major hepatobiliary centers who underwent surgical management for neuroendocrine liver metastasis (NELM) from 1985 to 2009. Major hepatectomy was performed in 45% of patients, and 14% underwent a second liver operation. Median survival was 125 months, with overall 5- and 10-year survival of 74%, and 51%, respectively. Disease recurred in 94% of patients at 5 years. Patients with hormonally functional NET who had R0/R1 resection benefited the most from surgery (*P* = 0.01). In a multivariate analysis, synchronous disease, nonfunctional NET hormonal status, and extrahepatic disease were independent predictors of worse survival (*P* < 0.05). Thus, while surgical resection for NELM is associated with prolonged survival, the majority of patients will develop recurrent disease. Patients with hormonally functional hepatic metastasis without prior extrahepatic or synchronous disease derive the greatest survival benefit from surgical management.

### 2.2. Radiofrequency Thermal Ablation (RFA)

Mazzaglia et al. [7] reported a prospective trial of 80 RFA sessions which was performed in 63 patients with NELM. Tumor types included 36 “carcinoid”, 18 pancreatic islet cell, and 9 medullary thyroid cancer. RFA was performed 1.6 years after the diagnosis of liver metastases. Median number of lesions treated was 6. The majority (49%) underwent 1 ablation session, and 14 (22%) had repeat sessions caused by disease progression. Fifty-seven percent of patients exhibited symptoms. One week postoperatively 92% of patients reported at least partial symptom relief, and 70% had significant or complete relief. Duration of symptom control was 11 months. Larger dominant liver tumor size and male gender adversely impacted survival (*P* < 0.05). Median survival times were 11.0 years after diagnosis of primary tumor, 5.5 years after diagnosis of NELM, and 3.9 years after first RFA. RFA, therefore, provides effective local control with prompt symptomatic improvement.

### 2.3. Liver Transplantation

If metastases are limited to the liver, orthotopic liver transplantation (OLT) is a viable treatment option [8]. OLT is currently offered to patients with unresectable metastases or for palliation of medically uncontrollable symptoms. Very few centers had reported experience representing more than 10 patients. Lehnert reviewed 103 cases who underwent OLT for metastases of NET in the largest review so far. Overall, 2-year and 5-year survival for all 103 patients was 60% and 47%, respectively, but recurrence-free 5-year survival did not exceed 24%. Three favorable prognostic factors were identified: age less than 50 years old, primary tumor location in lung or bowel, and pretransplant somatostatin therapy. In contrast, extensive abdominal operations were associated with poor prognosis. Thus, liver transplantation may be indicated in highly selected patients to provide immediate relief of otherwise intractable pain or hormone-related symptoms. OLT has no clear role in the routine treatment of patients with NET due to relatively high rates of tumor recurrence [9–11].

### 2.4. Transarterial Embolization (TAE)/Transarterial Chemoembolization (TACE)

Hepatic arterial embolization is commonly used as a palliative technique in patients with hepatic metastases who are not candidates for surgical resection. Hepatic artery embolization is based on the principle that tumors in the liver derive most of their blood supply from the hepatic artery, whereas healthy hepatocytes derive most of their blood supply from the portal vein. Embolization response rates are measured either by a decrease in hormonal secretion or by radiographic regression and are generally greater than 50% [12, 13]. In one of the largest series of 81 patients underwent embolization or chemoembolization for tumors labeled as “carcinoids” (likely of intestinal origin), the median duration of response was 17 months, and the probability of progression-free survival at 1, 2, and 3 years was 75%, 35%, and 11%, respectively [12].

Objective tumor responses have been noted in 33% to 67% of patients (Table 1). The variation of objective tumor response is related to the heterogeneous nature of tumors, various combination of cytotoxic agents, uncontrolled concomitant use of somatostatin analogues, and the difference in hepatic tumor burden.

Recent research has investigated the use of ^90^Y radioembolization to treat unresectable NELM. Kennedy et al. [24] reported that imaging response demonstrated stable lesions in 22.7%, partial response in 60.5%, complete response in 2.7%, and progressive disease in 4.9% of patients with only mild associated toxicity. The median survival from time of treatment was 70 months. Objective respond rate (complete and partial response) were observed in 50% of patients in a prospective studies, in which 32 patients were treated with ^90^Y microspheres [25].

## 3. Systemic Therapies

### 3.1. Somatostatin Analogs

Most neuroendocrine tumors (>80%) express a high density of somatostatin receptors (SSTR 1–5). Native somatostatin has not been useful in clinical practice due to its short half-life (<2 minutes). In 1980, Bauer et al. synthesized a somatostatin analog called octreotide, constituting an octapeptide with 3 unnatural amino acids, whereby the compound became resistant to metabolic degradation and presented a half-life of 3 to 4 hours in circulation. This peptide binds with high affinity to SSTR2 and SSTR5 and, therefore, inhibits the secretion of peptides and amines from neuroendocrine cells. In an initial study, the subcutaneous administration of the somatostatin analog octreotide, administered at a dosage of 150 mg 3 times a day, improved the symptoms of “carcinoid” syndrome in 88% of patients [26].

Octreotide has been widely used in oncology for almost 3 decades and is the most effective drug in inhibiting clinical symptoms related to hypersecretion of amines and peptides in NET. A long-acting depot form of octreotide (octreotide LAR), which can be administered on a monthly basis, has gained popularity. Octreotide therapy results in remission or stabilization of tumor markers, such as serotonin and chromogranin A, in approximately 60% to 70% of patients [27, 28].

PROMID [29] is the first randomized prospective trial demonstrating a possible antitumor effect for octreotide LAR compared with a placebo in patients with well-differentiated neuroendocrine tumors of midgut origin. A total of 85 patients with inoperable or metastatic well-differentiated midgut neuroendocrine tumors (carcinoid tumor) were randomized to receive either octreotide LAR 30 mg monthly or placebo. Median time to tumor progression was significantly longer for patients receiving octreotide (14.3 versus 6 months). This study supports an antiproliferative effect in well-differentiated midgut carcinoid tumors, with stabilization being the most frequently observed therapeutic response. However, only less than 10% tumor mass in the liver along with resected primary tumors responded to treatment. There was no significant difference in time to tumor progression between octreotide LAR and placebo in patients with larger tumor burden. The authors concluded that newly diagnosed NET with a low hepatic tumor burden and resected primary tumor were candidates for treatment with octreotide LAR.

The high rate of somatostatin receptor expression in NETs provides the rationale for peptide receptor radionuclide therapy (PRRT) as a treatment modality for patients with inoperable or metastatic disease. Several radiolabeled somatostatin analogs have been developed to treat patients with somatostatin receptor-positive metastatic tumors. The most frequently used radionuclides include yttrium (^90^Y) and lutetium (^177^Lu), which differ from one another in terms of emitted particles, particle energy, and tissue penetration. ^90^Y emits *β*-radiation has a range of 12 mm, and ^177^Lu emits both *β*-radiation and *γ*-radiation and has a range of 2 mm. ^177^Lu-DOTA, Tyr^3^-octreotate has since been utilized in the treatment of over 500 patients with GEP-NETs. Efficacy results, reported for 310 patients and 89% (276/310) presented liver metastasis, suggest an overall tumor response rate of up to 30%. However, all PRRTs using yttrium (^90^Y) and lutetium (^177^Lu) are not randomized, prospective studies and majority of patients are carcinoid [30–33].

### 3.2. Cytotoxic Chemotherapy

In a Phase II/III study of 249 patients with advanced “carcinoid” tumors, patients were randomized to receive either streptozocin/5-FU or 5-FU/doxorubicin [34]. The response rates were 16% and 15.9%, respectively. Although there was a slightly longer survival time associated with streptozocin/5-FU (24.3 versus 15.7 months) in this trial, over one-third of the patients treated with streptozocin developed renal toxicity. Thus, streptozocin-based regimens are not recommended in the first-line treatment of metastatic “carcinoid” tumors.

However, patients with advanced PNET may respond well to treatment with streptozocin and other alkylating agents. In a randomized trial, the combination of streptozocin and doxorubicin was associated with an overall response rate of 69% and a survival benefit, with median overall survival of 2.2 years [19]. A retrospective analysis of 84 patients with either locally advanced or metastatic PNET receiving a three-drug regimen of streptozocin, 5-FU, and doxorubicin showed that this regimen was associated with an overall response rate of 39% and a median survival of 37 months [21].

Temozolomide is an orally alkylating agent with a mechanism of action similar to streptozocin and dacarbazine. Retrospective studies suggest comparable progression-free survival (PFS) and overall survival (OS) between streptozocin and temozolomide-based regimens in patients with advanced PNET (Table 2). Prospective studies using temozolomide-based regimens in patients with advanced PNET are ongoing.

### 3.3. Targeted Therapies for Pancreatic Neuroendocrine Tumors

Studies of targeted therapies in PNET have, to date, focused primarily on inhibitors of the vascular endothelial growth factor (VEGF) or mammalian target of rapamycin- (mTOR) signaling pathways. Two phase III randomized studies suggested that treatment with these agents is associated with improvements in progression-free survival (PFS).

### 3.4. VEGF Pathway Inhibitors

Bevacizumab which targets VEGF and three tyrosine kinase inhibitors: pazopanib, sorafenib, and sunitinib—all with activity against VEGF receptor (VEGFR)—have been evaluated in prospective trials of patients with advanced PNET.

Bevacizumab is a recombinant humanized monoclonal antibody that binds to and neutralizes the biologic activity of human VEGF-A. In a randomized phase II study conducted at M.D. Anderson, 44 patients on stable doses of octreotide were randomly assigned to 18 weeks of treatment with bevacizumab or pegylated interferon alfa-2b (PEG IFN). A rapid and sustained decrease was observed in tumor perfusion following treatment with octreotide and bevacizumab, as measured in functional computed tomography. Clinical activity was evident by a response rate of 18% and an improved PFS rate at week 18 (95% versus 68%; *P* = 0.02). Bevacizumab therapy therefore provides an advantage in objective responses, reduction of tumor blood flow, and PFS in patients with carcinoid compared to PEG IFN treatment [37].

NETs frequently express VEGFR-2 and platelet-derived growth factor receptor receptor-*β* (PDGFR-*β*). Sorafenib, a small-molecule inhibitor of the VEGFR-2 and PDGFR-*β* tyrosine kinase domains, is a rational targeted therapy to be evaluated in NET. Hobday, et al. [38] reported a phase II study in 2007 ASCO using sorafenib in patients exposed to prior interferon and prior or concurrent octreotide at a stable dose. Patients received sorafenib 400 mg orally BID. A total of 93 patients were enrolled: (50 carcinoid and 43 PNET). For patients evaluable for the primary endpoint, 4 of 41 (10%) carcinoid patients and 4 of 41 (10%) PNET patients had a partial response (PR). There were 3 minor responses (MR = 20–29% decrease in sum of target lesion diameters) in carcinoid patients and 9 MRs in PNET patients and this led to PR + MR rate of 17% for carcinoid patients and 32% for PNET patients. Sorafenib at 400 mg orally twice a day in this study demonstrated modest activity in metastatic NET.

VEGF is a key driver of angiogenesis in PNET. Tissue from malignant PNET also shows widespread expression of PDGFRs *α* and *β*, stem-cell factor receptor (c-kit), and VEGFR-2 and VEGFR-3. Sunitinib inhibits these kinases and delays tumor growth in a RIP1-Tag2-transgenic mouse model of pancreatic islet-cell tumors by reducing endothelial-cell density and pericyte coverage of tumor vessels.

Sunitinib was evaluated in a multi-institutional phase II study enrolling 109 patients with advanced NET. Patients received sunitinib, administered orally at 50 mg once daily for 4 weeks, followed by a 2-week off period. Partial responses were observed in 2% of the carcinoid cohort and 16% of the PNET cohort [39]. Based on the encouraging response rate in this phase II study, an international randomized phase III study was conducted to confirm the activity of sunitinib in PNET. The study was discontinued prior to a planned interim analysis after enrollment of 171 patients, 86 of them received sunitinib, and 85 received placebo. The trial was terminated early because of the risk of serious adverse events, disease progression, and death among patients receiving placebo. The early discontinuation of the study precluded the definitive conclusion on differences in PFS durations between the treatment and placebo groups. Nevertheless, analysis of the available data demonstrated that treatment with sunitinib was associated with a remarkable median PFS of 11.4 months, as compared with 5.5 months for placebo (*P* = 0.0001) [35].

Pazopanib is an oral tyrosine kinase inhibitor of VEGFR, PDGFR, and KIT with both antiangiogenic and antitumoral activity. Pazopanib was evaluated in a prospective study enrolling 51 NET patients (29 with PNET and 22 with carcinoid) on stable doses of octreotide-LAR. Patients received pazopanib at a dose of 800 mg daily. The response rate among patients with PNET was 17%; no patients with carcinoid experienced a radiographic response (by RECIST). PFS rate at week 24 was 76% (80% PNET and 71% carcinoid). Median PFS times were 12.7 and 11.7 months, for carcinoid and PNET patients, respectively. Encouraging PFS durations in both carcinoid and PNET patients in this study suggested that treatment with pazopanib and octreotide seemed feasible and associated with tumor regression in patients with PNET [40].

### 3.5. mTOR Inhibitors

mTOR is a serine-threonine kinase that participates in the regulation of cell growth, proliferation, and apoptosis. Signaling through the PI3K/AKT/mTOR pathway leads to increased translation of proteins regulating cell-cycle progression and metabolism. This enzyme also mediates downstream signaling from a number of pathways, including the VEGF and insulin-like growth factor (IGF) signaling implicated in NET growth. The inhibition of mTOR prevents phosphorylation of key cell-cycle control proteins, leading to G1 growth arrest.

Temsirolimus and everolimus are rapamycin derivatives which were evaluated in NET. Weekly intravenous temsirolimus was associated with a response rate of 5.6% in a study of 37 patients with advanced progressive NET. Outcomes were similar between patients with carcinoid and PNET [41].

Everolimus was initially evaluated in a single-institution study, in which 30 patients with “carcinoid” tumors of intestinal origin and 30 with pancreatic PNET received doses of 5 or 10 mg daily plus depot octreotide (30 mg every 4 weeks). The overall tumor response rate in evaluable patients was 17% in carcinoid and 27% in PNET [42].

In a follow-up international phase II study (RADIANT-1), 160 patients with advanced PNET and evidence of RECIST-defined progression following chemotherapy were enrolled. In this nonrandomized study, treatment with everolimus was associated with an overall response rate of 4.4% and PFS duration of 16.7 months in those patients receiving octreotide. Among patients who did not receive octreotide, the response rate was 9.6%, and the PFS duration was 9.7 months [43].

A subsequent phase III study randomized 410 patients with progressive advanced PNET (RADIANT-3) to everolimus or placebo. This study demonstrated significant improvements in PFS (the primary endpoint) associated with everolimus as compared to placebo [(11 months versus 4.6 months (*P* < 0.0001)]. The overall tumor response rate associated with everolimus in this study was 5% [36]. A subgroup analysis of Japanese patients (23 patients received everolimus and 17 patients were in placebo arm) in the same RADIANT-3 study showed a significant 17 months improvement in PFS (19.45 versus 2.83 months) and an 81% risk reduction of progression or death (HR 0.19, 95% CI 0.08–0.48, *P* < 0.001) [44]. Phase III-randomized trials of biological targeted therapy in advanced neuroendocrine tumors are summarized in Table 3.

### 3.6. Combination of Target Therapies

Ongoing studies are evaluating combinations of targeted agents in patients with advanced neuroendocrine tumors. The combination of everolimus + bevacizumab was shown to be well tolerated and associated with antitumor activity (overall response rate 26%) in an initial phase II study enrolling patients with low- or intermediate-grade neuroendocrine tumors [43]. Other combination-advanced PNETs includes everolimus + temozolomide [45] and everolimus + octreotide [42].

## 4. Conclusions

Different therapeutic options have been employed for well-to-moderately differentiated NELM. Surgical resection of hepatic metastases or hepatic artery embolization can be helpful in patients with hepatic-predominant metastatic disease. Symptoms of hormonal excess, such as “carcinoid” syndrome and syndromes associated with functional PNET, can be effectively treated with somatostatin analogs. Treatment with the somatostatin analog octreotide has been shown to improve progression-free survival in patients with advanced midgut carcinoid tumors. Patients with NELM may also respond to treatment with streptozocin or temozolomide-based therapy but need to be reassessed using standard criteria of response. In patients with advanced PNET, randomized, placebo-controlled studies have recently demonstrated that treatment with the tyrosine kinase inhibitor sunitinib or with the mTOR inhibitor everolimus is associated with improved PFS. Initial phase II studies have also suggested activity associated with VEGF pathway and mTOR inhibitors in patients with neuroendocrine tumors of other origins including intestinal “carcinoids.” Future studies will likely define the utility of combinations of these agents in the treatments of patients with NELM.

## Figures and Tables

**Table 1:** Selected clinical studies of transarterial embolization (TAE)/transarterial chemoembolization (TACE) in metastatic NET.

Author (yr)	No. of patients	Disease	Therapy	Complete/partial response
Ruszniewski et al. [14] (1993)	24	Carcinoid/PNET	TACE	33%
Wängberg et al. [15] (1996)	40	Carcinoid	TAE	42.5%
Gupta et al. [12] (2003)	81	Carcinoid	TACE/TAE	67%
Strosberg et al. [16] (2006)	84	Carcinoid/PNET	TAE	48%
Marrache et al. [17] (2007)	38	Carcinoid/PNET	TACE/TAE	37%
Ho et al. [18] (2007)	33	Carcinoid/PNET	TACE/TAE	46%

**Table 2:** Selected clinical trials of cytotoxic chemotherapy in advanced PNET**.

Regimen	No. of patients	Tumor response rates (%)	Median PFS month	Complete/partial response	Author (yr)
Prospective studies					
Chlorozotocin	33	30	17	18	Moertel et al. [19] (1992)
STZ + 5FU	33	45	14	16.8	
STZ + DOX	36	69	18	26.4	
DTIC	50	34	NR	19.3	Ramanathan et al. [20] (2001)
Retrospective studies					
STZ + 5FU + DOX	84	39	18	37	Kouvaraki et al. [21] (2004)
TMZ various chemotherapy	53	34	13.6	35.3	Kulke et al. [22] (2009)
TMZ + Capecitabine	30	70	18	NR	Strosberg et al. [23] (2010)

**Several of the early studies often assessed tumor response and PFS by clinical and not imaging parameters.

PFS: progression-free survival, STZ: streptozocin, 5FU: 5-flurouracil, DOX: doxorubicin, DTIC: dacarbazine, TMZ: temozolomide, NR: not reported.

**Table 3:** Selected randomized trials of targeted therapy in advanced neuroendocrine tumors.

Regimen	No. of patients	Tumor response rates (%)	Median PSF (months)	% of NELM	Author (yr)
*PNET *					
Sunitinib 37.5 mg po qd	86	9	11.4 (*P* < 0.001)	71 (61/86)	Raymond et al. [35] (2011)
Placebo (+ best supportive care)	85	0	5.5	54 (46/85)	
Everolimus 10 mg po qd	207	5	11 (*P* < 0.001)	92 (190/207)	Yao et al. [36] (2011)
Placebo (+ best supportive care)	203	2	4.6	92 (187/203)	
Everolimus 10 mg po qd					
Everolimus 10 mg po qd + Bevacizumab 10 mg/kg every other week	GALGB 80701	Ongoing			
*Carcinoid*					
Octreotide LAR	42	2	14.3 (*P* < 0.001)	83 (35/42)	Rinke et al. [29] (2009)
Placebo	43	2	6.0	88 (38/43)	
Everolimus + octreotide LAR	187	Radiant-2 accrual completed	Final report pending		
Placebo + octreotide LAR	191				
Octreotide + bevacizumab	SWOG S0518				
Octreotide + placebo					
